# Accelerating forest tree breeding by integrating genomic selection and greenhouse phenotyping

**DOI:** 10.1002/tpg2.20048

**Published:** 2020-08-20

**Authors:** Filipe C. Alves, Kelly M. Balmant, Marcio F. R. Resende, Matias Kirst, Gustavo de los Campos

**Affiliations:** ^1^ Department of Epidemiology and Biostatistics Michigan State University East Lansing MI 48824 USA; ^2^ School of Forest Resources and Conservation University of Florida Gainsville FL 32611 USA; ^3^ Plant Molecular and Cellular Biology Graduate Program University of Florida Gainsville FL 32611 USA; ^4^ Horticulture Science Department University of Florida Gainsville FL 32611 USA; ^5^ Department of Statistics and Probability Michigan State University East Lansing MI 48824 USA; ^6^ Institute for Quantitative Health Science and Engineering Michigan State University East Lansing MI 48824 USA

## Abstract

Breeding forest species can be a costly and slow process because of the extensive areas needed for field trials and the long periods (e.g., five years) that are required to measure economically and environmentally relevant phenotypes (e.g., adult plant biomass or plant height). Genomic selection (GS) and indirect selection using early phenotypes (e.g., phenotypes collected in greenhouse conditions) are two ways by which tree breeding can be accelerated. These approaches can both reduce the costs of field‐testing and the time required to make selection decisions. Moreover, these approaches can be highly synergistic. Therefore, in this study, we used a data set comprising DNA genotypes and longitudinal measurements of growth collected from a population of *Populus deltoides* W. Bartram ex Marshall (eastern cottonwood) in the greenhouse and the field, to evaluate the potential impact of integrating large‐scale greenhouse phenotyping with conventional GS. We found that the integration of greenhouse phenotyping and GS can deliver very early selection decisions that are moderately accurate. Therefore, we conclude that the adoption of these approaches, in conjunction with reproductive techniques that shorten the generation interval, can lead to an unprecedented acceleration of selection gains in *P. deltoides* and, potentially, other commercially planted tree species.

**Abbreviations**
BLUE: best linear unbiased estimate
BLUP: best linear unbiased predictor
DNA: deoxyribonucleic acid
GI
: generation interval
GBLUP: genomic BLUP
GS: genomic selection
LD: linkage disequilibrium
MAF: minor allele frequency
MTG‐F: multi‐trait genomic prediction model using field phenotypes
MTG‐GF: multi‐trait genomic model integrating greenhouse and field phenotypes
PI: phenotypic index based on greenhouse phenotypes
SNP: single nucleotide polymorphism
STF‐G: single‐trait GBLUP using field phenotypes
TRN‐TST: the training‐testing partitions.

## 1 INTRODUCTION

Poplars (*Populus* spp.) and their hybrids are considered an ideal short‐rotation woody crop (SRWC). These species and/or hybrids have multiple applications in the industry, providing reliable wood products, veneer, pulp and paper, excelsior, chemicals, and feedstocks for cellulosic energy and the biofuels industry (Maier et al., 2019). Poplars can also offer important environmental applications such as phytoremediation (Isebrands & Richardson, 2014; Stanton, Neale, & Li, 2010). Forest species are also one of the main carbon sinks on the planet (Bellassen & Luyssaert, 2014). In particular, because of their fast growth, poplar species have been recommended as efficient carbon dioxide sequesters above and below ground. Studies have reported that soil carbon tends to increase between 9% (in heavy soils) to 62% (in sandy soils) under poplar plantations relative to annual crops (Isebrands & Richardson, 2014).

The genus *Populus* includes 29 species distributed across the northern hemisphere (Eckenwalder, 1996). The genetic improvement of *Populus spp*. has been done primarily by combining first‐generation interspecific hybridization with reciprocal recurrent selection for the parental species (Stanton et al., 2010). According to Stanton et al. (2010), among all existing species in this genus, the ones with most interest for breeding and testing in the United States and Europe are *P. nigra* (black poplar), *P. maximowiczii*, *P. trichocarpa* (western balsam poplar or black cottonwood), and *P. deltoides* (eastern cottonwood). Among them, *P. deltoides* is the most used species as parental for *Populus* hybridization programs (Isebrands & Richardson, 2014).

Artificial selection is one of the main ways by which forest's productivity and environmental services can be improved; however, breeding forest species is a laborious, expensive, and slow process. Genomic selection (GS; Meuwissen, Hayes, & Goddard, 2001) has been adopted in a myriad of plant species. This approach can be particularly useful to accelerate the breeding of forest tree species, reducing or eliminating the period required for an individual to express its full genetic potential (e.g., Grattapaglia & Resende, 2011; Resende et al., 2012a, 2012b, 2012c; Grattapaglia et al., 2018).

Greenhouse phenotyping can be used to measure phenotypes (e.g., growth traits) at the very early stages of the growth cycle; these phenotypes can be used for screening and early selection of large numbers of genotypes. Therefore, indirect phenotypic selection based on greenhouse phenotypes is another approach by which the generation interval can be reduced, and selection intensity can be increased.

Important factors affecting the accuracy of GS include the trait heritability, the size of the training set, the extent of linkage disequilibrium in the genome and the genotyping density, and the strength of genomic relationships between training and testing individuals (e.g., de los Campos et al., 2013; Goddard & Hayes, 2009). On the other hand, the accuracy of indirect phenotypic selection relies on the strength of genetic correlations between the traits used for selection and the selection objective (e.g., Falconer & Mackay, 1996).

In tree breeding, the recent history of selection has not been as intense as in other agricultural species. Low levels of linkage disequilibrium (LD) and weak genetic relationships can limit the accuracy of GS. Under those circumstances, correlated traits measured in the candidates of selection can improve prediction accuracy. Therefore, we hypothesize that integrating traditional GS with early phenotyping can lead to improvements in prediction accuracy relative to the use of either of the strategies alone. Therefore, in this study, we used a data set comprising DNA genotypes and longitudinal measurements of growth (plant height) collected in a greenhouse (from week 1 to 15) and in the field (at years 1 to 5) to evaluate the potential impact of integrating large‐scale greenhouse phenotyping with traditional GS.

**Core Ideas**

- Greenhouse measurements of plant height (week 15) are genetically correlated with plant height at year 5.
- Indirect phenotypic selection using early greenhouse phenotyping achieves good selection accuracy.
- Integrating early phenotyping into genomic selection models can increase prediction accuracy.
- Genomic selection using early greenhouse‐ and field ‐phenotyping can accelerate tree breeding without compromising accuracy.

## 2 MATERIALS AND METHODS

### 2.1 Phenotypic data

The phenotypic data (*n* = 473 genotypes) was generated using a collection of *P. deltoides* (eastern cottonwood) genotypes developed and maintained at the University of Florida. Genotypes were sampled in 15 states in central, southern, and eastern United States. Four clones of each of the genotypes were grown under greenhouse conditions, and another set of 473 clones from the same genotypes were grown under field conditions in Citra, Florida. Plant height was measured weekly for the first nine weeks of growth (W.1, …, W.9) in the greenhouse and biweekly from week 9–15 (W.11, W.13, W.15). In the field, plant height was measured yearly from years 1 to 5 (Y.1, …, Y.5). In both conditions, the experiments were conducted using complete randomized block designs, with four blocks, with one biological replicate (clone) per genotype per block.

The greenhouse and field phenotypes were pre‐adjusted using mixed models that accounted for the experiment (intercept), block (random), rows and columns (for the field data, both as random), bench (random, only in the greenhouse data), and genotypes (random). Separate mixed models were fitted to each of the time‐points (W.1, …, W.15, Y.1, …, Y.5). The adjusted phenotypes used for downstream analyses were the best‐unbiased estimate (BLUE) of each replicate.

### 2.2 Genotyping

A total of 92K SNPs were available for each individual (details about the genotyping procedure are available in Fahrenkrog et al., 2017b). Quality control procedures were performed considering a call rate lower than 0.75 and MAF lower than 0.05. Missing genotypes were imputed using the mean of each SNP. After the quality control procedures, a total of 68,885 markers were available for further analyses.

### 2.3 Prediction models

We considered various models to predict plant height in the field, including single‐ and multi‐trait models, as well as indirect phenotypic prediction using greenhouse phenotypes. Genomic analyses were carried out using a Genomic BLUP approach (GBLUP; VanRaden, 2008).

The single‐trait GBLUP (STG‐F) had the following specification:

(1) $$\;y_{t}=\;1\mu_{t}\;+\;Zu_{t}+\varepsilon_{t}$$

where $y_{t}$ is the vector of adjusted phenotypes at time‐point *t*, **1** is a vector of 1s, $\mu_{t}$ is an intercept, $u_{t}$ is the vector of genomic effects of the clones at time‐point *t*, **Z** is the design matrix for the genetic effects, and $\varepsilon_{t}$ is the vector of error terms at time‐point *t*. Both genomic effects and errors were assumed to be multivariate normal (MVN): $u_{t}∼MVN\left(0,K\sigma_{u_{t}}^{2}\right)$ and $\varepsilon_{t}∼MVN\left(0,I\sigma_{\varepsilon_{t}}^{2}\right)$, where $\sigma_{u_{t}}^{2}$ is the additive genetic variance of plant height at time *t*, and **K** is genomic relationship matrix obtained from (centered) SNP genotypes (**X**), $K\;=\frac{{\mathrm{XX}}^{\prime }}{tr({\mathrm{XX}}^{\prime })/n}$, and $\sigma_{\varepsilon_{t}}^{2}$ is the error variance at time *t*. Separate single‐trait GBLUP models were fitted to plant height at years 1 to 5.

For multi‐trait models (incorporating plant height data from *T* time points), we used GBLUP model of the form:

(2) $$\;\left[\begin{array}{c} y_{1}\\ \vdots \\ y_{T} \end{array}\right]=\left[\begin{array}{ccc} I_{1} & \cdots & 0\\ \vdots & \ddots & \vdots \\ 0 & \cdots & I_{T} \end{array}\right]\;\left[\begin{array}{c} \mu_{1}\\ \vdots \\ \mu_{T} \end{array}\right]+\left[\begin{array}{ccc} Z_{1} & \cdots & 0\\ \vdots & \ddots & \vdots \\ 0 & \cdots & Z_{T} \end{array}\right]\left[\begin{array}{c} u_{1}\\ \vdots \\ u_{T} \end{array}\right]+\left[\begin{array}{c} \varepsilon_{1}\\ \vdots \\ \varepsilon_{T} \end{array}\right].$$

The standard multi‐trait genomic model considering all time points (MTG‐GF) contains the random effects of the genotypes and the error terms had MVN distributions with (co)variances across time‐points: ${\left({u^{\prime }}_{1},\ldots ,{u^{\prime }}_{T}\right)}^{\prime }∼MVN\left(0,G⊗K\right)$, where **G** is a (T×T) unstructured covariance matrix of the genetic effects across time‐points, and ${\left({\varepsilon^{\prime }}_{1},\ldots ,{\varepsilon^{\prime }}_{t}\right)}^{\prime }∼MVN\left(0,R⊗I\right)$, where **R** is the covariance matrix of the error terms. For models involving joint analyses of greenhouse and field data, **R** was block‐diagonal, $R\;=\left[\begin{array}{cc} R_{\mathrm{GH}} & 0\\ 0 & R_{F} \end{array}\right]\;$ where **R**_GH_ and **R**_F_ are unstructured (co)variance matrices for traits measured in the greenhouse and field conditions, respectively. We also considered a multi‐trait GBLUP for field data only (MTG‐F), in this case, $R\;=\;R_{F}$.

We also considered indirect phenotypic selection using a phenotypic selection index (PI) derived from the greenhouse phenotypes:

(3) $$\;PI_{i}=By_{GH,i}\;$$

where $PI_{i}$ is a (5×1) vector of predicted field phenotypes for the *i^th^* clone, $y_{GH,i}$ is the vector of the (adjusted) greenhouse phenotypes for the same clone, and **B** is a matrix of weights which were derived using Selection Index methodology (Smith, 1936; Hazel, 1943): $B\;=G_{F,GH}P_{GH}^{-1}\;$. Here, $G_{GH,F}$ is a matrix of genetic (co)variance between the field and greenhouse phenotypes, and $P_{GH}=G_{GH}\;+R_{GH}$ is the phenotypic covariance matrix of the greenhouse phenotypes. These (co)variances were estimated using the standard multi‐trait GBLUP model (MTG‐GF).

### 2.4 Assessment of selection accuracy

Following Lopez‐Cruz et al. (2020), we focused on genetic (rather than phenotypic) prediction accuracy. The genetic accuracy of a predictor ($\hat{y}$, e.g., a genomic prediction in the testing set, or a phenotypic index) is equal to the product of the square‐root of the heritability of the predictor, times the genetic correlation of the predictor and the predictand (*y*, any of the field phenotypes) (Falconer & Mackay, 1996), that is

(4) $$Acc\;\left(\hat{y},y\right)=\sqrt{h_{\hat{y}}^{2}}\;\times r_{g_{\hat{y},y}}$$

**FIGURE 1:**
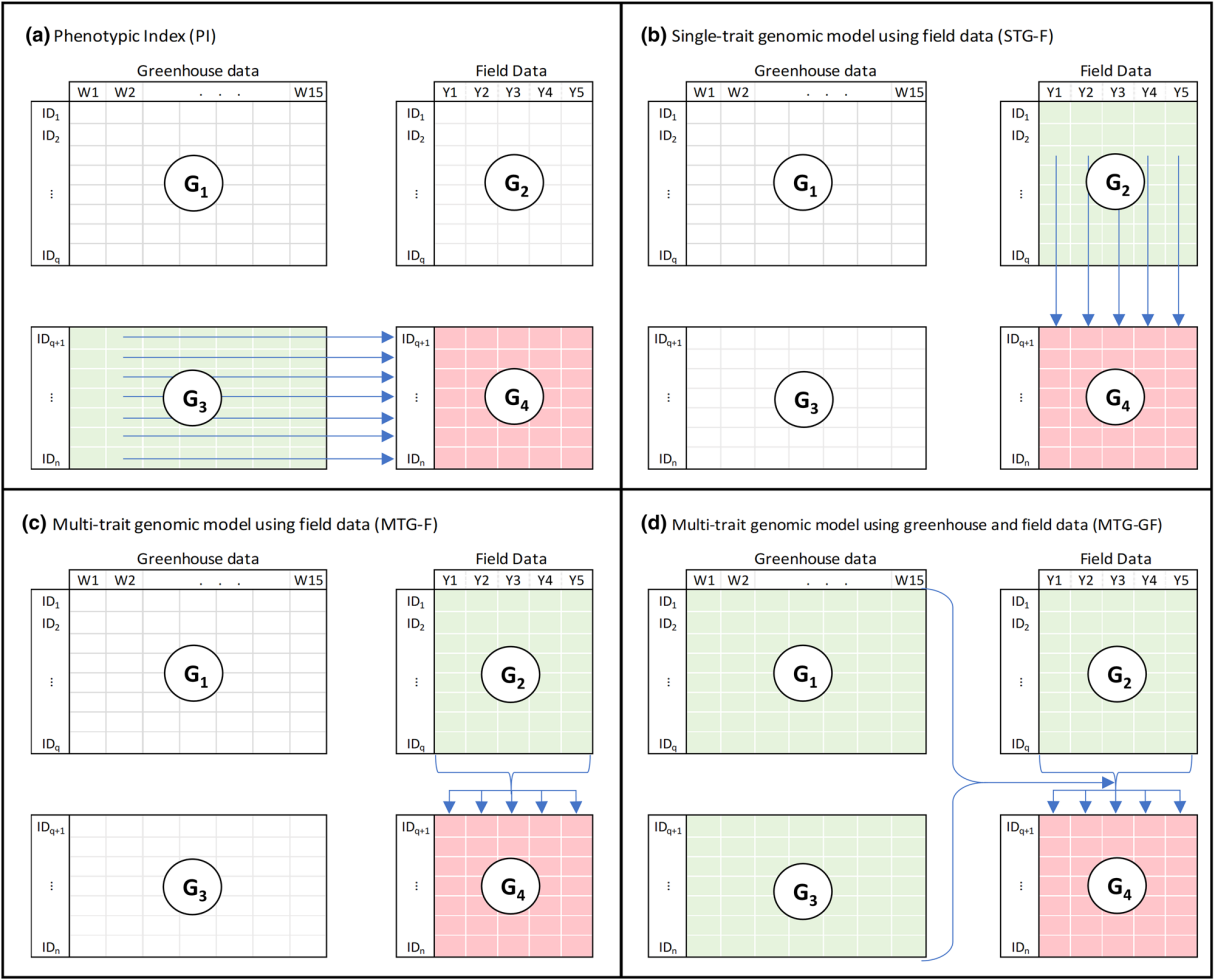
Four prediction methods and the corresponding training and testing sets used. (a) Phenotypic index, estimated based on the phenotypes collected in the greenhouse, (b) Single‐trait genomic prediction model using field phenotypes (STG‐F), (c) Multi‐trait genomic model (MTG‐F), and (d) Multi‐trait genomic model combining greenhouse and field genotypes (MTG‐GF). Blocks colored in green, red, and white represent the information used for training, testing, and not used for neither training nor testing, respectively. W: week; Y: year, ID: individual, G: subset groups of the data

To evaluate the impact of integrating genomic data and greenhouse phenotyping on the selection accuracy, we fitted four different models (Figure 1): The training sets for each model are described below:

- The **Phenotypic index** (PI, Equation 3) predicts field phenotypes using greenhouse information (G3).
- **Single‐trait genomic models** (STG‐F, the model of Equation 1 fitted to each of the field phenotypes separately) uses only the field data (G2) as the training set.
- The **Multi‐trait genomic models** (MTG‐F, the model of Equation 2 fitted only to the field phenotypes) used the same training data as the STG‐F (G2) but considered all field traits jointly.
- Finally, we considered a **Multi‐trait genomic model** that used all the greenhouse (G1 and G3) and field data (G2) for model training (Equation 2, denoted as MTG‐GF for the selection purposes).

The testing set (G4, in Figure 1) consisted of 25% of the field data and was the same across prediction methods. We replicated 100 times the training‐testing partitions (TRN‐TST) presented in Figure 1. For each TRN‐TST, we estimated $h_{\hat{y}}^{2}$ and $r_{g_{\hat{y},y}}$ by fitting a two‐trait GBLUP model with $\left(\hat{y},y\right)$ as traits, where $\hat{y}$ and *y*, are respectively, the predicted values and the observed phenotypes of the individuals in the composing testing set. Later, we derived the selection accuracy (Equation 4) for each of the methods. Differences in selection accuracy across methods were assessed using Tukey's Honest Significant difference applied to the 100‐point estimates available.

### 2.5 Software

The single and multi‐trait GBLUP models were fitted using the BGLR (Pérez & de los Campos, 2014) and MTM R‐packages (de los Campos & Grüneberg, 2016). For each model, we ran 30,000 cycles of a Gibbs sampler; the first 5,000 samples were discarded as burn‐in and the remaining 25,000 were thinned at an interval of 5. Further details of the implementation can be found in Supplemental Note S1.

## 3 RESULTS

**FIGURE 2:**
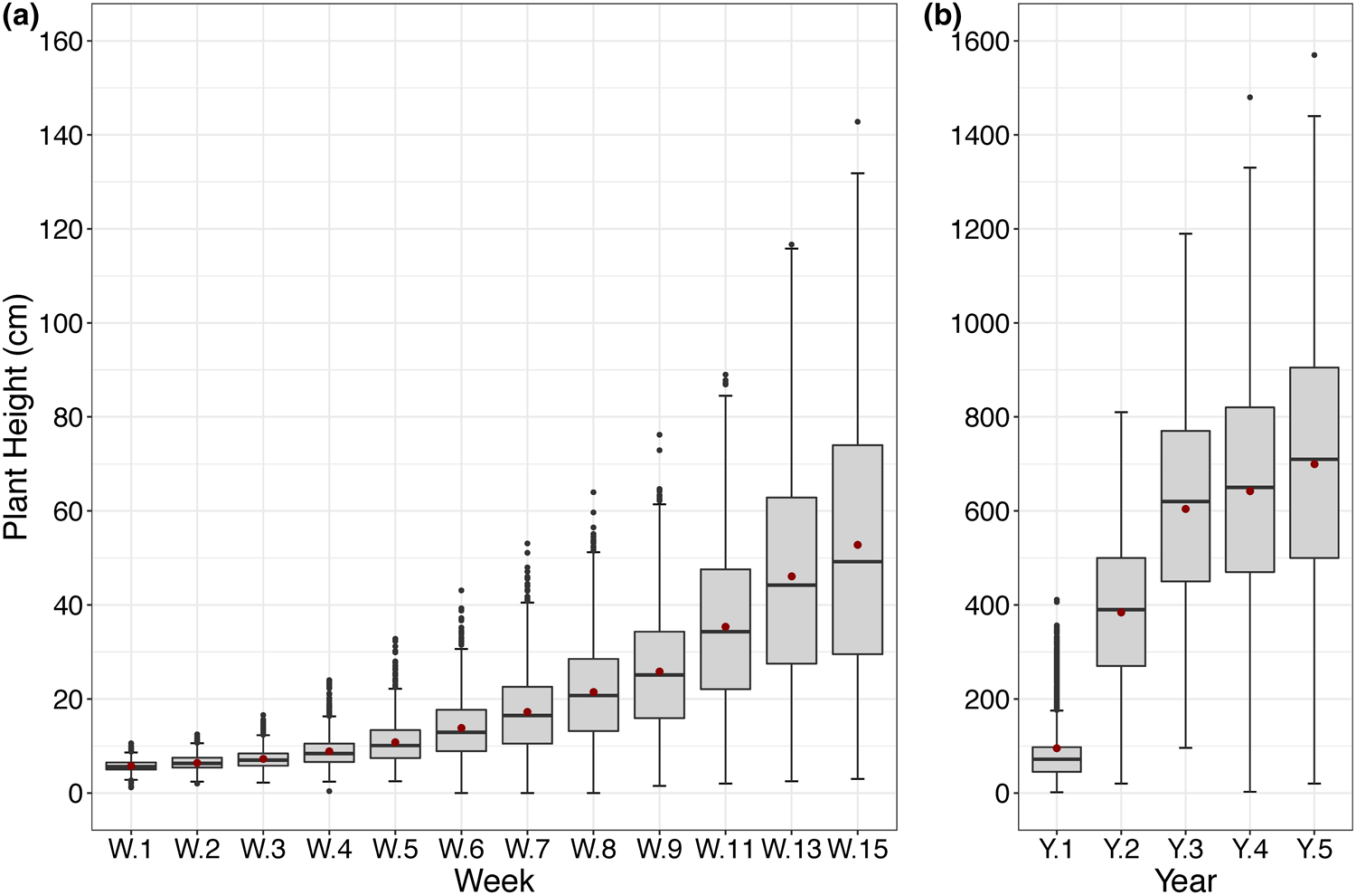
Plant height on greenhouse (a) and field (b) conditions for *P. deltoides*. The red points represent the means. W.1‐15 and Y.1‐5: Weekly greenhouse and yearly field measurements

Plant height in the greenhouse increased from an average of 5.73 cm in W.1 to 52.77 cm in W.15 (Figure 2a). In the field, the average height increased faster in the first three years, achieving an average plant height of 604.34 cm at Y.3, and then increased at lower rates between Y.3 and Y5, achieving an average height of 699.60 cm at Y.5 (Figure 2b).

**FIGURE 3:**
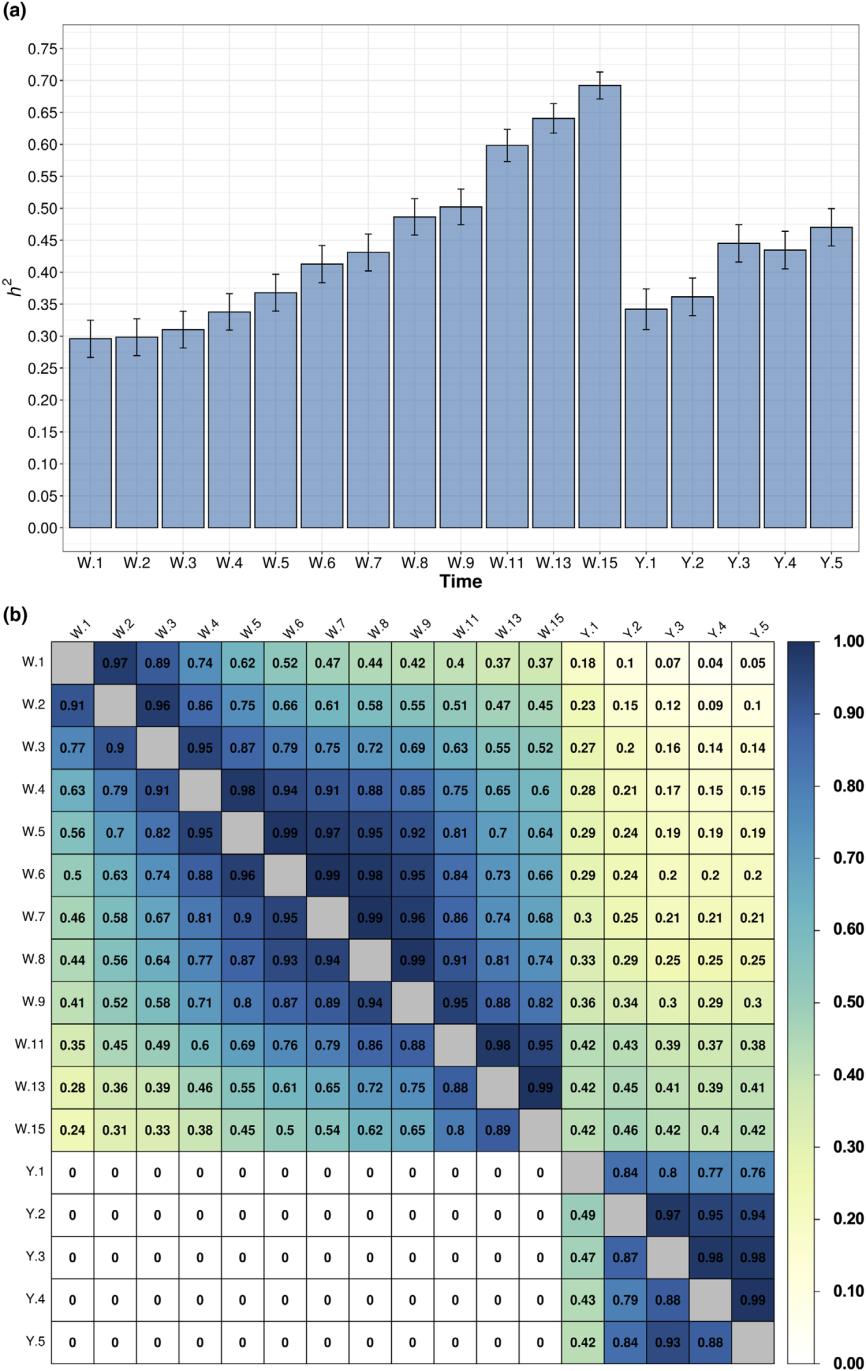
Genetic and environmental parameters for plant height in *P. deltoides*. (a) Heritability estimates (posterior standard deviation in the vertical interval). (b) Genetic (upper‐triangle) and environmental (lower‐triangle) correlations. W: Weekly greenhouse measurements; Y: Yearly field measurement

Narrow‐sense heritability (*h* ^2^) estimates obtained with the multi‐trait GBLUP ranged from ∼0.296 (W.1) to ∼0.692 (W.15) in the greenhouse (Figure 3a; Supplemental Table S1), and between ∼0.25 (Y.2) to ∼0.47 (Y.5) in the field. We also estimated the broad‐sense heritability (*H* ^2^) using a mixed‐effects model with the random effect of the genotype without assuming an additive model (Supplemental Table S1). As expected, the broad‐sense heritabilities were higher than the narrow‐sense heritabilities, but the differences between the two were modest (∼10%), with a ratio $h^{2}/H^{2}$ ranging from 0.911 (Y.4) to 0.974 (W.13) (the ratio for Y.1 was 1.03 which could happen due to estimation errors in both *h* ^2^ and *H* ^2^). The genetic correlations between consecutive measurements of plant height in the greenhouse were (as one would expect for traits measured weekly or bi‐weekly) very high, decreasing smoothly with the time‐lag between measurements (Figure 3b). Importantly, the genetic correlations between plant height measured at W.11 to W.15 with plant height in the field were moderate (e.g., 0.42 between plant height at W.15 and plant height at Y.5).

**FIGURE 4:**
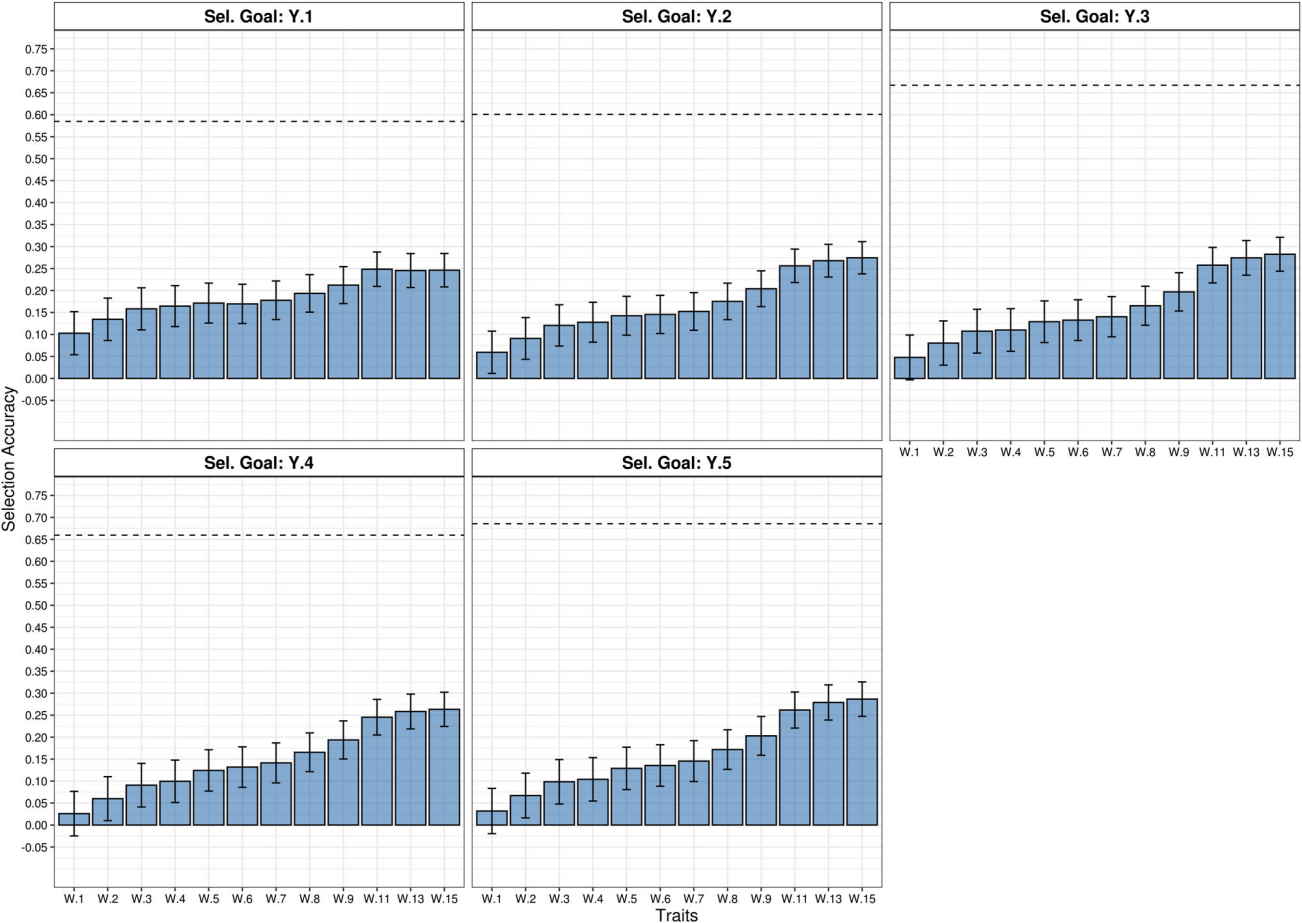
Selection accuracy for field plant height (years 1–5, Y.1, Y.2, Y.3, Y.4, and Y.5) of *P. deltoides* achieved by indirect phenotypic selection based on individual greenhouse measurements (weeks 1–15, W.1‐W.15). The vertical bars represent the posterior standard deviation of the estimated accuracy; the dashed line is the square root of the narrow‐sense heritability of the selection goals (Y.1, Y.2, Y.3, Y.4, Y.5)

The accuracy of the indirect selection for weekly greenhouse measurements (estimated based on the genetic parameters presented in Figures 3a and 3b) was low for the very early greenhouse measurements (Figure 4). However, later plant height measurements (e.g., W.15) had selection accuracies that represented up to ∼42% of the square‐root of the narrow‐sense heritability (black dashed lines in the plots) in the last two years of field evaluations.

**FIGURE 5:**
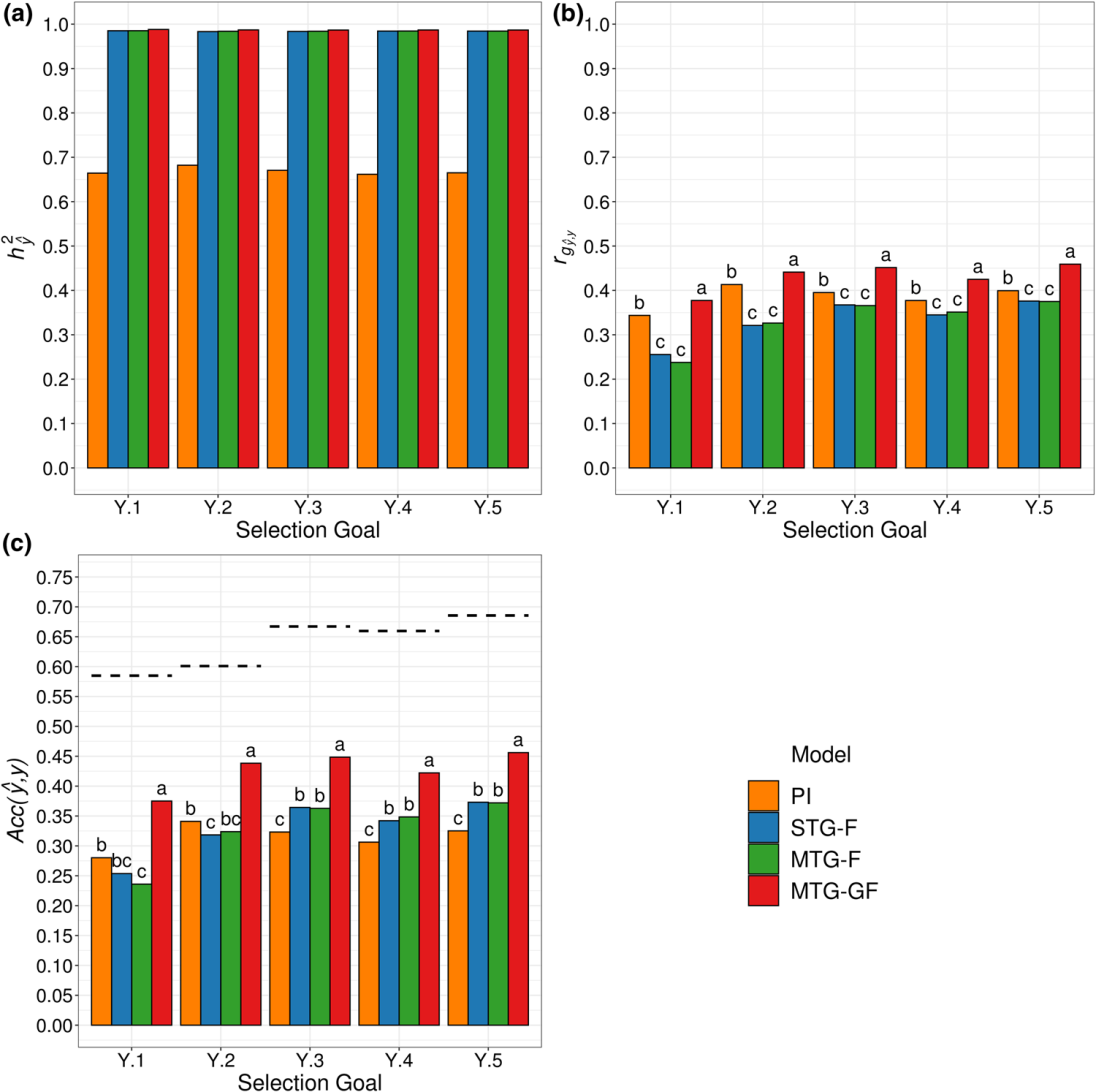
Average heritability of the predictions (**a**, $h_{\;\hat{y}}^{2}$), mean genetic correlation between the predictions and the selection goals (**b**, $r_{g_{\hat{y},y}}$), and selection accuracy (**c**, $Acc(\hat{y},y)$) by model in a *P. deltoides* population. PI: Phenotypic index; STG‐F: Single‐trait genomic model considering the field phenotypes; MTG‐F: Multi‐trait genomic model considering only field phenotypes; MTG‐GF: Multi‐trait genomic model considering greenhouse and field phenotypes. Y: Year in which the trait was measured in the field. Within selection goal, letters indicate that the differences are statistically significant at the 0.05 level in the Tukey's Honest Test. The dashed lines represent the square root of the trait's narrow‐sense heritability (selection accuracy of the direct selection) for the respective year of field evaluation

As expected, the heritability of the genomic predicted values was very close to one (Figure 5a); this happens because genomic predictions are a linear combination of SNP‐genotypes, and replicates (clones) of the same genotype have identical predicted values. The PI (phenotypic index) had lower heritability than predictions from GBLUP models (Figure 5a); however, the PI had a higher genetic correlation with plant height in the field than predictions derived from models that did not incorporate phenotypes of the selection candidates (i.e., those solely based in field data, STG‐F, MTG‐F, Figure 5b). All the models that did not use phenotypes measured in the selection candidates (STG‐F and MTG‐F) showed lower $r_{g_{\hat{y},y}}$ estimates than the models that use greenhouse phenotypes measured in the candidates of selection (PI and MTG‐GF). Therefore, the higher genetic correlations obtained with PI and the MTG‐GF seem to be a direct consequence of the information provided by the greenhouse phenotypes measured on the candidates of selection.

Among all the models tested, the one integrating field and greenhouse phenotypes (MTG‐GF) achieved both high heritability and high genetic correlation with the selection goals. Consequently, this model achieved the highest selection accuracies (Figure 5c). The selection accuracies of this model were at least 21% higher than those of the other methods. The relative efficiency (i.e., the ratio of $Acc(\hat{y},y)$ to the square‐root of the heritability of the target trait) of the MTG‐GF was 0.73 for Y.2 and, and ranged from 0.64 to 0.67 for the other years (Supplemental Table S2).

Implementing the best performing model (MTG‐GF) requires having a reference population that has been genotyped and has greenhouse (G1) and field phenotypes (G2), plus SNP‐genotypes and greenhouse phenotypes for the candidates of selection (G3). One can imagine a situation where greenhouse phenotypes are only available for the candidates of selection. This will be the case if one has a reference data set for GS that only integrates field phenotypes and supplements this information with greenhouse data on the candidates of selection. Therefore, to consider this possibility, we also fitted the MTG‐GF model using G2 and G3 as training data. The genetic prediction accuracies achieved by this model were slightly smaller than the ones achieved by the MTG‐GF model trained with G1, G2, and G3 data; thus, suggesting that there are marginal benefits of having a reference population with both greenhouse and field phenotypes (Supplemental Table S2).

## 4 DISCUSSION

The genetic improvement of crops is one of the main approaches by which agricultural output can be increase while preserving natural resources. Annual response to selection (RS) depends on the amount of genetic variation ($\sigma_{g}$, the genetic standard deviation of the selection goal), selection intensity (*i*), the accuracy of selection (i.e., the correlation between the selection goal and the selection criteria, $Acc\left(\hat{y},y\right)\;=\;Cor\left(\hat{y},y\right)$), and the generation interval (GI, e.g., Falconer & Mackay, 1996), thus:

$$RS\;=\frac{i\times Acc\left(\hat{y},y\right)\;\times \sigma_{g}}{GI}\;$$

Tree breeding can be an expensive and slow process because testing trees in field conditions is costly and GI is long. Reducing the GI is one of the main ways by which tree breeding can increase response to selection (Grattapaglia & Resende, 2011; Grattapaglia et al., 2018; Harfouche et al., 2012). Early phenotypic selection is often used to reduce the GI, but it is generally not applied before half of the age that the tree is harvested (e.g., 3–6 years, depending on the end‐use of the wood product). Genomic selection (GS) and indirect phenotypic selection using very early (i.e., weeks instead of years) phenotypic measurements are alternative ways by which generation interval could be shortened (e.g., Resende et al., 2012a). In this study, we evaluated these two approaches, as well as the potential benefit of integrating them.

**Traditional genomic selection** uses a reference data set comprising genotypic and phenotypic data to estimate SNP effects for the selection goal (e.g., phenotypes in Y.5). Genomic predictions are then derived using DNA genotypes of the selection candidates and the estimated effects. In this study, we tested single‐trait (STG‐F) and multi‐trait (MTG‐F) genomic prediction models. Using these models, one could imagine reducing the generation interval to the minimum allowed by the reproductive system. The relative efficiencies (i.e., the selection accuracy relative to the square‐root of the trait's narrow‐sense heritability) achieved by these models ranged between 0.51 and 0.54 (Supplemental Table S2) for plant height in years 4 and 5.

The accuracy of GS models trained without phenotypes of the candidates of selection is highly dependent on family relationships (Habier, Fernando, & Dekkers, 2007), and on long‐spanning LD (or, conversely, small effective population size). In this population, the effective population size is large (e.g., Fahrenkrog et al., 2017a), genomic relationships are weak (Supplemental Figure S1a), and LD has a fast decay (Supplemental Figure S1b). These features, which are common in many tree populations, distinguish trees from animals (e.g., dairy cattle) and annual crops (e.g., wheat) where GS has been most effective. Large effective population size and short span of LD can impose limits on the selection accuracy that can be achieved by GS models. Therefore, higher marker density and large training populations are required to achieve and to maintain selection accuracies (Grattapaglia & Resende, 2011; Isik, 2014). This is especially important in the early generations in which the effect of selection on LD is not very strong.

While it is clear that family relationships contribute significantly to prediction accuracy when models are trained with family data, several studies in humans (e.g., Kim, Grueneberg, Vazquez, Hsu, & de los Campos, 2017; Lello et al., 2018) have shown that GS models can predict complex traits even when training and testing populations are distantly related (e.g., genomic relationships smaller than 0.003). The relative contribution of family relationships to prediction accuracy may depend on the extent of the genomic relationships in the data used to train models. The inspection of the genomic relationship matrix in our study (Supplemental Figure S1a) shows that most materials are distantly related, but there are some clusters of materials with intermediate relationship (∼0.2). These relationships can clearly contribute to prediction accuracy, but the extent of genomic relationships in our data set is low, compared to what one expects in a breeding population. Therefore, considering the critical role that LD and family relationships play in prediction accuracy, we expect that more accurate genomic predictions could be obtained when the models discussed in this study are applied to breeding populations. However, in such a context, model re‐training will be needed to account for changes in LD patterns that occur across generations.

**Early indirect phenotypic selection using greenhouse phenotypes** is another way by which the generation interval could be shortened. Our results indicate that the genetic correlation between growth phenotypes measured in the greenhouse at weeks 13 and 15 and those measured in the field at years 3–5 is moderate (0.39‐0.42, Figure 3b). Furthermore, combining multiple greenhouse phenotypes into a selection index (PI) for plant height at Y.1‐Y.5, lead to a relative efficiency of ∼0.480 (Supplemental Table S2). This efficiency is comparable to that observed in GS models based only on field phenotypes (e.g., MTG‐F).

**To further improve selection accuracy, we integrated field phenotypes with greenhouse phenotypes of the candidates of selection** into a multi‐trait GS model. This model (MTG‐GF) leveraged both genetic similarity between training and testing genotypes, as well as genetic correlations between traits, which lead to the borrowing of information between traits within individuals. This model achieved the best predictive performance with a relative efficiency of selection very similar to that observed for the direct mass phenotypic selection (Supplemental Table S2). The increase in prediction accuracy that we observed when including correlated phenotypes evaluated in the candidates of selection (e.g., compare accuracies of the MTG‐F and MTG‐GF) is comparable to the gains in accuracy previously reported in annual crops using multi‐environment GS models (e.g., Burgueño, de los Campos, Weigel, & Crossa, 2012; Cuevas et al., 2017; Dias et al., 2018; Jarquín et al., 2014; Lopez‐Cruz et al., 2015).

Using MTG‐GF for selection requires refitting the model any time a new batch of greenhouse phenotypes becomes available; this may be logistically challenging. Alternatively, as a more practical approach, one could consider averaging genomic prediction with a PI. For that, one can use the following three‐step approach: *(i)* estimate of SNP effects with a standard genomic model (e.g., MTG‐F or STG‐F) trained with field data only; *(ii)* derive PI from the greenhouse phenotypes weights calibrated from variance components estimates; and *(iii)* obtain a final index as a weighted average of the two predictors by using ${\;\mathrm{PI}}_{w}=\;W\;\times \;\mathrm{PI}+\left(1-W\right)\;\times \hat{Y}$, where PI and $\hat{Y}$ are the phenotypic index and predicted value obtained using MTG‐F (or STG‐F) of the genotype, respectively and W is the relative weight assigned to the PI. We quantified the selection accuracy of this approach using weights on the PI ranging from 0 (MTG‐F) and 1 (PI). Our results (Supplemental Table S3) suggest that placing equal weight on the genomic prediction and the PI yields the most accurate index. Moreover, the prediction accuracy of the resulting index was not (statistically significantly) different than the accuracy of the full MTG‐GF.

To the best of our knowledge, this is the first study involving GS in *P. deltoides* using longitudinal traits. Recently, Campbell, Walia, and Morota (2018) and Momen, Campbell, Walia, and Morota (2019) shown the importance of modeling longitudinal variation for the prediction of the projected shoot area during the first thirty days of plant development in rice. In our study, due to the growth biology of tree species, we considered a much longer growth period that spanned from the first week of growth in the greenhouse to the 5^th^ year in the field. To model longitudinal trajectories, we used a multi‐trait model where plant heights measured at different time‐points were considered correlated traits. Our model used an un‐structured genetic covariance matrix for genetic effects. The estimated covariance patterns were relatively smooth with respect to time, as expected, nearby time points have higher genetic correlations than time points that were further apart in time. This result suggests that a potential alternative approach for model the growth could be to use random regression models (e.g., splines) similar to the ones used to model lactation curves (Jamrozik & Schaeffer, 1997; Liu, Zhang, Schaeffer, Yang, & Zhang, 2006; Schaeffer, 2004) or growth in beef cattle (Meyer, 1998, 2000, 2005). However, spline models impose covariance patterns that may be too restrictive (Meyer & Kirkpatrick, 2008), especially for field conditions for which the number of time‐points is small and the time‐span between points is large. Furthermore, given the observed growth patterns, the environmental differences between greenhouse and field trials, and the different timescale (weeks versus years), it seems that different splines would be needed for the greenhouse and the field data.

Most forest tree species require a long period between seed germination and flowering. For most poplar species, such as *P. deltoides*, this period may extend for more than five years. This delay in flowering limits the advantages that can be obtained by combining very early selection and genomic approaches that were considered in this study. However, for many forest tree species, accelerating flowering is not an insurmountable challenge, and has been addressed using specific environmental and horticultural treatments. For instance, treatment of juvenile tree with triazole growth regulators such as paclobutrazol has often been used to induce early flowering in *Eucalyptus* (Griffin, Whiteman, Rudge, Burgess, & Moncur, 1993). Alternatively, transgenic approaches have been applied to manipulate genes in the reproductive developmental pathway and induce early flowering in poplar (Hoenicka, Lehnhardt, Nilsson, Hanelt, & Fladung, 2014, 2016; Hsu, Liu, Luthe, & Yuceer, 2006). Alternative methods, such as speed breeding (Watson et al., 2018), may also be applicable in the future, although the large stature of trees may limit the use of this approach.

Therefore, there are many techniques that could result in flowering within the first year following seed germination, thus reducing GI by a factor of potentially 70% (e.g., from 5 to 1.5 years). Thus, the integration of field and greenhouse phenotyping into a GS model (MTG‐GF) could lead to an increase in response to selection, relative to the direct selection on Y.5 plant height, of about 121% (0.665/0.3, where 0.665 is the relative efficiency of the MTG‐GF method, see Supplemental Table S2). In addition to the impacts on the genetic gains that can be achieved through a reduction in GI, both greenhouse phenotyping and GS may enable an expansion of the number of genotypes tested, leading to an increase in selection intensity that will further improve response to selection.

In our study, we applied genomic prediction to a collection of “wild” genotypes of *P. deltoides*. This is very close to scenarios faced by breeding programs of forest trees in which the genetic differentiation between breeding and natural populations is still small (Isik, 2014). To evaluate impacts on long‐term selection, we focused on additive models and used the narrow‐sense heritability as a benchmark. However, breeding programs in poplar can rely on clonal propagation of (intra and interspecific) F1 hybrids, which often involve *P. deltoides* lines due to its fast growth rate (Stanton et al., 2010). Clonal propagation leverages both additive and non‐additive genetic effects. In our dataset, the broad‐sense heritability of plant height was about 10% higher than the narrow sense heritability (see Supplemental Table S1). This suggests that prediction accuracy for clonal propagation could be improved even further by considering non‐additive effects. Recent studies have shown moderate improvements in accuracy when considering non‐additive effects in annual crops (Alves et al., 2019; Philipp et al., 2016) and forest trees (Almeida Filho et al., 2016; Muñoz et al., 2014; Tan, Grattapaglia, Wu, & Ingvarsson, 2018). Moreover, some studies have shown that non‐additive genetic effects play an important role on the phenotypic expression of growth traits in trees (Tan et al., 2018). Therefore, models incorporating non‐additive effects could be used for both further improving prediction accuracy and optimal mate allocation (e.g., Varona, Legarra, Toro, & Vitezica, 2018).

## 5 CONCLUSIONS

Genomic selection and indirect mass phenotypic selection using greenhouse phenotyping can yield moderate selection accuracies for plant height at year 5. Combining these two technologies can further improve selection accuracies. Therefore, the use of GS and early phenotyping of selection candidates could, if paired with adequate propagation/reproduction techniques, lead to a substantial improvement in response to selection in *P. deltoides*.

## CONFLICT OF INTEREST

The authors declare that there is no conflict of interest.

## Supporting information

Supplemental Figure S1: Genomic relationship among individuals (a), genome‐wide linkage disequilibrium and LD decay (b) in a P.
Supplemental Table S1: Narrow (*h* ^2^) and road‐sense (*H* ^2^) heritabilities (posterior standard deviation) of plant height under greenhouse (“W”, weeks) and field (“Y”, year) conditions.
Supplemental Table S2: Accuracy of selection (relative efficiencies) and training sets by model. Low case‐letters represent the groups obtained by a Tukey's Honest Test at 5%
Supplemental Table S3: Accuracy of selection of weighted index (PI_*w*_) and MTG‐F. Low case‐letters represent the groups obtained by a Tukey's Honest Test at 5% significance.

## Data Availability

The data that support the findings of this study are openly available in Mendeley Data at http://doi.org/10.17632/62rw2j25gx.2. The file contains the corrected phenotypes and the genomic relationship matrix used to perform the statistical analyses used in this paper.
