# Supplementary material for: Accelerating forest tree breeding by integrating genomic selection and greenhouse phenotyping
Source: Plant Genome. 2020 Aug 20;13(3):e20048. doi: 10.1002/tpg2.20048 (PMC12807299; doi:10.1002/tpg2.20048)
Supplement: Supplementary file 1 [file TPG2-13-e20048-s001.docx]

**Supplemental Materials**

**Title:** Accelerating forest tree breeding by integrating genomic selection and greenhouse phenotyping

**Authors:** Filipe Couto Alves, Kelly M. Balmant, Marcio F. R. Resende Junior, Matias Kirst, Gustavo de los Campos

**8 pages, 3 supplementary tables, 1 supplementary figure, and 1 supplementary note (code used to run the analyses)**

**Supplemental Figure S1:** Genomic relationship among individuals (**a**), genome-wide linkage disequilibrium and LD decay (**b**) in a *P. deltoides* population.

**Supplemental Table S1:** Narrow ($h^{2})$ and road-sense ($H^{2}$) heritabilities (posterior standard deviation) of plant height under greenhouse (“W”, weeks) and field (“Y”, year) conditions.

| **Trait** | $\boldsymbol{h}^{\boldsymbol{2}}$ | $\boldsymbol{H}^{\boldsymbol{2}}$ |
| --- | --- | --- |
| W.1 | 0.296 (0.029) | 0.316 (0.027) |
| W.2 | 0.298 (0.029) | 0.315 (0.026) |
| W.3 | 0.310 (0.029) | 0.332 (0.026) |
| W.4 | 0.338 (0.028) | 0.368 (0.026) |
| W.5 | 0.368 (0.029) | 0.402 (0.026) |
| W.6 | 0.413 (0.029) | 0.446 (0.025) |
| W.7 | 0.431 (0.029) | 0.462 (0.025) |
| W.8 | 0.486 (0.028) | 0.508 (0.024) |
| W.9 | 0.502 (0.028) | 0.522 (0.024) |
| W.11 | 0.598 (0.025) | 0.614 (0.021) |
| W.13 | 0.641 (0.023) | 0.658 (0.020) |
| W.15 | 0.692 (0.021) | 0.711 (0.018) |
| Y.1 | 0.342 (0.032) | 0.332 (0.029) |
| Y.2 | 0.361 (0.029) | 0.386 (0.026) |
| Y.3 | 0.445 (0.029) | 0.479 (0.026) |
| Y.4 | 0.435 (0.029) | 0.477 (0.026) |
| Y.5 | 0.47 (0.029) | 0.512 (0.025) |

**Supplemental Table S2:** Accuracy of selection (relative efficiencies) and training sets by model. Low case-letters represent the groups obtained by a Tukey’s Honest Test at 5% significance.

|  | **Training set^*^** | | | **Selection Goal** | | | | |
| --- | --- | --- | --- | --- | --- | --- | --- | --- |
| **Model** | **G_1_** | **G_2_** | **G_3_** | **Y.1** | **Y.2** | **Y.3** | **Y.4** | **Y.5** |
| PI |  |  |  | 0.280 (0.479) c | 0.341 (0.568) c | 0.323 (0.484) d | 0.306 (0.464) d | 0.325 (0.474) d |
| STG-F |  |  |  | 0.254(0.434) cd | 0.318 (0.530) d | 0.364 (0.546) c | 0.342 (0.519) c | 0.373 (0.544) c |
| MTG-F |  |  |  | 0.236 (0.404) d | 0.324(0.539) cd | 0.363 (0.544) c | 0.348 (0.528) c | 0.372 (0.543) c |
| MTG-GF |  |  |  | **0.375 (0.641) a** | **0.438 (0.730) a** | **0.449 (0.672) a** | **0.422 (0.640) a** | **0.456 (0.665) a** |
| MTG-GF^1^ |  |  |  | 0.333 (0.569) b | 0.416 (0.693) b | 0.426 (0.638) b | 0.403 (0.612) b | 0.425 (0.620) b |

**^*^**Groups present in the training set (Figure 1);

^1^ MTG-GF fitted considering only the evaluation of newly developed lines in the greenhouse (S3) and old lines in the field (S2)

**Supplemental Table S3:** Accuracy of selection of weighted index ($PI_{w}$) and MTG-F. Low case-letters represent the groups obtained by a Tukey’s Honest Test at 5% significance.

| **W*** | **Y.1** | **Y.2** | **Y.3** | **Y.4** | **Y.5** |
| --- | --- | --- | --- | --- | --- |
| 0.0 | 0.238 h | 0.325 g | 0.364 d | 0.349 e | 0.374 e |
| 0.1 | 0.269 g | 0.355 ef | 0.389 c | 0.372 d | 0.397 d |
| 0.2 | 0.303 e | 0.387 d | 0.415 b | 0.396 c | 0.422 c |
| 0.3 | 0.330 cd | 0.413 c | **0.436 a** | 0.416 ab | 0.443 b |
| 0.4 | 0.348 bc | **0.429 ab** | **0.448 a** | **0.428 a** | **0.456 ab** |
| 0.5 | **0.355 ab** | **0.435 a** | **0.449 a** | **0.430 a** | **0.458 a** |
| 0.6 | 0.351 bc | **0.429 ab** | **0.438 a** | **0.421 a** | **0.447 ab** |
| 0.7 | 0.338 bcd | 0.414 bc | 0.418 b | 0.401 bc | 0.426 c |
| 0.8 | 0.320 de | 0.392 d | 0.390 c | 0.373 d | 0.396 d |
| 0.9 | 0.300 ef | 0.367 e | 0.357 d | 0.340 e | 0.362 e |
| 1.0 | 0.280 fg | 0.341 fg | 0.323 e | 0.306 f | 0.326 f |
| MTG-GF | **0.375 a** | **0.438 a** | **0.449 a** | **0.422 a** | **0.456 ab** |

*Weight used to derive the index. The weighted index was obtained by: $PI_{w}=W\times PI+(1-W)\times\hat{Y}_{MTG-F}$, where $\mathrm{PI}$ and $\hat{Y}_{MTG-F}$ are the phenotypic index and predicted value obtained using MTG-F of the genotype, respectively.

**Supplemental Note S1:** Code used to fit the genomic models

### **Loading data**

```
### Library

library(MTM)

### Data

load("Data.RData")
str(y) ## Phenotype's file structure
str(G) ## Genomic relationship matrix
Y<-y[,3:19]
Y<-scale(Y)
Yfield<-Y[,15:19]
nIter<-1000
burnIn<- 100
nfolds=4

### Number of traits by growing conditions

nGreenMeas<-sum(grepl("wk",colnames(Y))) ## number of measurements in greenhouse
nFieldMeas<-sum(grepl("F",colnames(Y))) ## number of measurements in field
nT<- nGreenMeas+nFieldMeas  ## Number of traits

### Creating the testing-set 75%/25$ training-testing partition
 set.seed(123456)
 folds<-c(sample(rep(1:nfolds,each=round(nlevels(y$UFID)/nfolds))),1)
 LevelsID_CV<-data.frame(ID=levels(y$UFID),folds) ## Linking IDs to folds

## Testing set
 TestSet<-y$UFID%in%LevelsID_CV$ID[LevelsID_CV$folds==4]

### Generating the ZGZ' matrix for the genomic analyses (First Step)
Z<-model.matrix(~UFID-1,data=y)
ZAZp<-Z%*%G%*%t(Z)
```

**#First Step:**

### **#a) Fitting the recursive MTG-GF model (greenhouse plus field phenotypes)**

```
## Masking the phenotypes of the testing set
yNA<-Y
yNA[TestSet,13:17]<-NA

### ETA for the fully recursive residuals(no residual covariance between greenhouse ### and field phenotypes) #

  M1<-matrix(FALSE, ncol=nGreenMeas , nrow=nGreenMeas)
  M2<-matrix(FALSE, ncol=nFieldMeas,nrow=nGreenMeas)
  M3<-matrix(FALSE, ncol=nGreenMeas,nrow=nFieldMeas)
  M4<-matrix(FALSE, ncol=nFieldMeas,nrow=nFieldMeas)
  M1[lower.tri(M1)]<-TRUE
  M4[lower.tri(M4)]<-TRUE
  M<-rbind(cbind(M1,M2),cbind(M3,M4))

  Resid<-list(type="REC",M=M,S0 = rep(1,nT),df0 = rep(1, nT),var=100)


#### ETA for unstructured genetic effects

df=nT+4
Cov=cov(Y,use='pairwise.complete') ## Phenotypic covariance between phenotypes
covStr=list(type="UN",df0=df,S0=(df-nT-1)*Cov/2)
RANEF=list(list(K=ZAZp, COV=covStr))

### Fitting the MTG-GF model

fm<- MTM(Y = yNA, K = RANEF, resCov = Resid, nIter = nIter, burnIn = burnIn, thin = 5)
```

### **#b) Fitting the MTG-F model (considering only field phenotypes**)

```
set.seed(123456)

yNA<-Yfield
yNA[TestSet,] <-NA

### ETA for unstructured genetic effects

df=nFieldMeas+4
Cov=cov(Yfield,use='pairwise.complete') ## Phenotypic covariance between phenotypes
covStr=list(type="UN",df0=df,S0=(df-nT-1)*Cov/2)
RANEF=list(list(K=ZAZp, COV=covStr))

### Fitting the MTG-F model

fm<- MTM(Y = yNA, K = RANEF, resCov = Resid, nIter = nIter, burnIn = burnIn, thin = 5)
```

### **#Saving outputs (any MTM model)**

```
### Recovering outputs

yHat<-fm$YHat ## Predicted values
G<-fm$K[[1]]$G ## Trait's Genetic covariance matrix (T by T)
R<-fm$resCov$R ## Residual covariance matrix
P<-G+R ## Phenotypic covariance matrix
```

### **#c) Estimating phenotypic indexes (PI) of greenhouse phenotypes (needs the covariance # obtained in the MTG-GF model)**

```
 E=1:12 ## Columns of Y containing the greenhouse phenotypes
 Index<-matrix(ncol=nFieldMeas,nrow=nrow(Y))
 Ps<-P[E,E] ## Phenotypic covariance matrix for the greenhouse phenotypes
 Pi<-solve(Ps)

  for(j in 1:5){
    k=c(13:17)[j] ## Indicate the year for which the index will be estimated (column    in Y)
    Ps<-P[1:12,1:12]
    Y1<-Y[,E]
    GCGreenField<-matrix(G[k,E],nrow=1) ## Genetic Covariance between the field
                                        ## and the greenhouse phenotypes
    Betas<-GCGreenField%*%solve(Pi)
    Index[,j]<-Y1%*%t(Betas)
  }
```

**#Second Step:**

### **#a) Estimating the genetic parameters of the predictions - genetic correlations between # indexes (predictions or phenotypic index) and observed phenotypes, heritabilities of the # predictions, and accuracy of selection. This example considers a 75%TRN-25%TST # cross-validation for the MTG-GF model.**

```
yPred<-yHat[TestSet,13:17] ### Example for the MTG-GF
                             ### One can use : yPred<-Index[TestSet,] for PI
                             ### or yPred<-yHat[TestSet,] for the MTG-F

#### Generating ZGZ for the testing set

 IDs<-droplevels(y$UFID[TestSet])
 Z<-model.matrix(~IDs-1)  ### Z matrix for the testing set

 Test_ID<-rownames(G)%in%unique(IDs)
 G_t<-G[Test_ID,Test_ID]
 ZGttZp<-Z%*%G_t%*%t(Z)  ### ZGZ' for the testing set

#### Estimating the selection accuracy (Acc), genetic correlation (GGcor) and #### heritability (H2CV

 Acc<-GGcor<-H2CV<-matrix(nrow=5,ncol=1)

  for(s in 1:5){
   pred<-cbind(yPred[,s],Yfield[TestSet,s])
   pred<-scale(pred)
   df0=ncol(pred)+4
   S0=cov(pred,use="pairwise.complete")*(df0-2)
   Resid<-list(type="UN",S0 =S0,df0 = rep(df0, 2))
   covStr=list(type="UN",df0=df0,S0=S0)
   RANEF=list(list(K=ZAZt,
              COV=covStr))

   fm<- MTM(Y = pred,K = RANEF,resCov = Resid,nIter = nIter,
                  burnIn = burnIn,thin = 5)

## Retrieving genetic (GG) and residual (Res) covariance matrices

   GG<-fm$K[[1]]$G
   Res<-fm$resCov$R

## Estimating genetic parameters (heritabilities of the predictions -H2CV-   ## and genetic correlation (GGcor)

   GGcor[s,1]<-cov2cor(GG)[1,2]
   H2CV[s,1]<-GG[1,1]/(GG[1,1]+Res[1,1])

## Estimating the accuracy of selection (r=sqrt(h2CV)*Gcor):

   Acc[s,1]<-sqrt(GG[1,1]/(GG[1,1]+Res[1,1]))*cov2cor(GG)[1,2]
}
```
